# The identity of three South American “smiliine” treehoppers (Hemiptera, Membracidae) and related taxonomic changes, including description of a new genus in Thuridini

**DOI:** 10.3897/zookeys.678.10340

**Published:** 2017-06-06

**Authors:** Stuart H. McKamey

**Affiliations:** 1 USDA/ARS Systematic Entomology Laboratory, 10th St. & Constitution Ave., PO Box 37012, Washington, DC, 20013-7012 USA

**Keywords:** New genus, new combination, new placement, Smiliinae, Smiliini

## Abstract

Based on examination of holotypes or interpretation of original descriptions, four taxonomic changes are proposed for South American species erroneously placed in the tribe Smiliini: *Flynnia*, **gen. n.** (Thuridini) and *F. fascipennis* (Funkhouser), **comb. n.** from Bolivia; *Antianthe atromarginata* (Goding), **comb. n.** from Ecuador; *Amastris pilosa* (Funkhouser), **comb. n.** from Peru; and *Thelia planeflava* Fairmaire from Brazil to Polyglyptini *incertae sedis*, **new placement.**

## Introduction

Previously, McKamey and Wallace (2015) evaluated the true identities of South American records of the Nearctic tribe Telamonini, which had all been the result of errors in labeling, in published distribution, or species that belonged to other subfamilies. Four species of the related tribe Smiliini (Smiliinae) (*sensu* Wallace 2011) were also described from South America: *Ophiderma fascipennis* Funkhouser from Bolivia, *Atymna pilosa* Funkhouser from Peru, Cyrtolobus (Atymna) atromarginata Goding from Ecuador, and *Thelia planeflava* Fairmaire (Telamonini but most recently placed in the genus *Ophiderma* of Smiliini) from Brazil. Smiliines are principally Nearctic, with numerous species occurring in the mountains of Central America, and throughout their range usually feed on oaks (*Quercus* sp.). Because oaks drop out of the flora in northern Colombia, the existence of true smiliines in South America was highly suspect. Examination of three holotypes and one original descriptions of these species revealed their identities as listed below.

## Methods

Holotypes of three species were in the National Museum of Natural History, Washington, DC (USNM). The holotype of the other species could not be located, so its placement is inferred from original descriptions. Morphological terminology follows Deitz (1975).

To examine the holotype of *Ophiderma fascipennis*, the abdomen and right metathoracic leg were removed from the specimen and treated with 8-10% KOH for 45 minutes, rinsed with water and then transferred to glycerin for further dissection and examination. After examination, the dissected male genitalia and metathoracic leg were stored in a microvial with fresh glycerin and pinned below the specimen. The head and pronotum, as a unit, were also separated so that the mesonotum could be examined, to possibly infer nymphal structure. This latter separated body part was pointed on the same pin as the head and pronotum.

All images were captured with a Microvision system and Cartograph 8.0.6 automontage software and adjusted in Adobe Photoshop.

## Results

### Tribe Amastrini Goding

#### Amastris pilosa

Taxon classificationAnimaliaHemipteraMembracidae

(Funkhouser) comb. n.

Figs 1–4

Atymna pilosa Funkhouser 1919: 273 [sp. n.].

##### Material examined.

**Figures 1–4.**
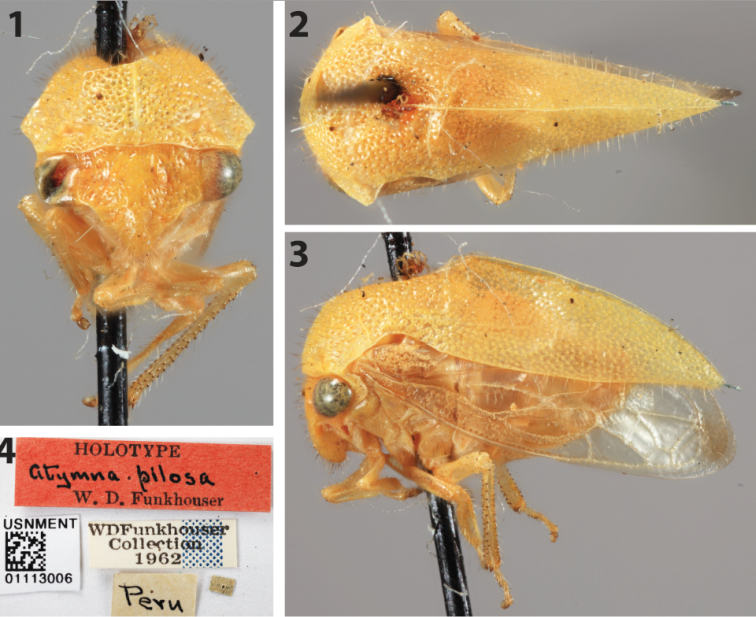
*Amastris pilosa* (Funkhouser) female holotype in anterior, dorsal, and lateral views, respectively, and labels.

*Atymna pilosa* female holotype, Peru (USNM; Figs 1–4).

##### Discussion.

The holotype, which has its pronotum partially damaged dorsoanteriorly, exhibits all features of *Amastris* Stål, including the forewing veins R and M initially fused then strongly divergent (a character diagnostic for the tribe). Within the tribe only *Amastris* and *Erosne* Stål have tectiform pronota, and *Erosne* has a sturdier pronotum with a yellow lateral margin.

### Tribe Polyglyptini Goding

#### Polyglyptini

Taxon classificationAnimaliaHemipteraMembracidae

incertae sedis, new placement

Thelia planeflava Fairmaire, 1846: 306. [sp. n.] Brazil.
Heranice planeflava ; Funkhouser 1927: 317.
Ophiderma planeflava ; Goding 1929: 277.

##### Discussion.

No holotype or other specimen was located. Fairmaire’s (1846) description of *T. planeflava* translates to: “Prothorax projecting backward, very little elevated, rugosely punctate throughout the head; entirely yellow; base of the abdomen a little orange; forewings hyaline, with the internal margin slightly coated.” He reported it as 6 mm long.

In the mid 1800’s, Fairmaire’s period, the concept of *Thelia* Amyot & Serville contained many unrelated taxa that have since been referred to other tribes and subfamilies. Fairmaire’s (1846) publication is a good example, with three of his species now belonging to *Hypheodana* Metcalf (Darninae: Darnini), *Carynota* Fitch (Smiliinae: Telamonini), and *Heranice* Stål (Smiliinae: Polyglyptini). His placement of his fourth, Brazilian species, *planeflava* in *Thelia*, therefore, offers no clues to it’s true identity. It is also unfortunately not illustrated, as were the other species.

In his catalogue, without explanation, Funkhouser (1927) moved *planeflava* from *Thelia* to *Heranice*, and Goding (1929) moved it to *Ophiderma* Fairmaire. Schmidt (1931) discussed *planeflava* and ultimately included it in his key to *Heranice*, restating Fairmaire’s original description.

Based on Fairmaire’s description, the species’ length, and reported distribution, it is unlikely to be any of the aforementioned genera; *Thelia*, *Carynota*, and *Ophiderma* have Nearctic distributions (and further, *Ophiderma* feeds on oaks, which do not occur in Brazil). *Heranice* are larger and apparently confined to high elevations in the Andes Mountains, and *Hypheodana* are brown.

Polyglyptini often have the anterior region of the forewing coriaceous and punctate, which may be what Fairmaire considered “coated” and, while no entirely yellow species are known, most Polyglyptini genera have a slightly elevated pronotum that extends backward over the body.

### Tribe Thuridini Deitz

#### Flynnia gen. n.

Taxon classificationAnimaliaHemipteraMembracidae

http://zoobank.org/FCB8F8E3-36A3-4B4A-889C-B2CC7D40654E

##### Type species.

*Ophiderma fascipennis* Funkhouser, 1919: 274.

##### Description.

**Figures 5–9.**
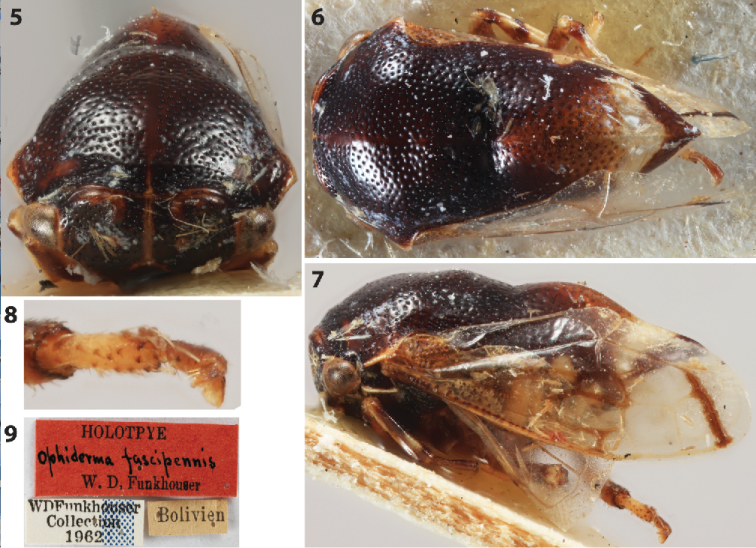
*Flynnia fascipennis* (Funkhouser) male holotype. **5–7** Habitus in anterior, dorsal, and lateral views, respectively **8** Right metathoracic tarsus **9** labels.

**Figures 10–12.**
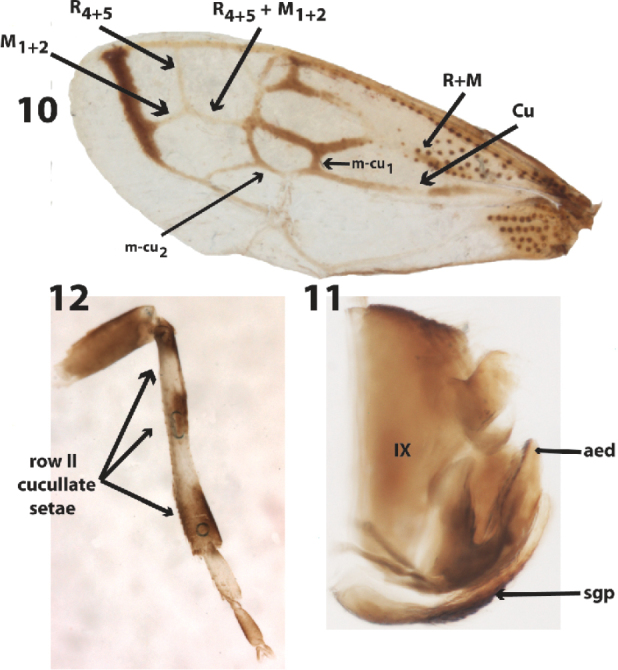
*Flynnia fascipennis* (Funkhouser) male holotype. **10** left forewing **11** pygofer, subgenital plate, aedeagus **12** Right metathoracic leg. *aed*, aedeagal shaft, *sgp*, subgenital plate.

Head. Vertex with dorsal margin sinuate. highest between ocelli and eyes; ocelli slightly closer to inner margin of eyes than to each other; frontoclypeus evenly rounded ventrally; rostrum attaining abdomen. Thorax. Posterior process of pronotum smooth and finely punctate throughout (Figs 5–7), weakly sinuous in lateral view, slightly overlapping forewings in repose. Mesonotum bare, lacking vestigial scoli. Forewing (Fig. 10) with veins R and M fused basally and strongly divergent near middle of wing, veins R_4+5_ and M_1+2_ confluent for a short distance and very strongly divergent more distally, 2 m-cu crossveins present, 1^st^ near mid length of wing. Hind wing without r-m crossvein, veins R_4+5_ and M_1+2_ confluent for short distance and divergent beyond, anal vein unbranched. Metathoracic leg (Fig. 12) without cucullate femoral setae, tibia without cucullate setae in row 1, very few in row II (where the leg is more strongly sclerotized, as indicated by arrows, Fig. 12), first tarsomere with about eight cucullate setae scattered on plantar surface (Fig. 8). Abdomen. Lacking dorsal protrusions or smooth fossae.

##### Distribution.

Bolivia and Ecuador.

##### Etymology.

The genus is feminine and named for Dawn Flynn for her contributions to our knowledge Neotropical treehoppers.

##### Discussion.

Many of the aforementioned features are diagnostic for the tribe Thuridini as listed by Deitz (1975): posterior process of pronotum slightly overlapping forewings; forewing with veins R and M fused basally and strongly divergent near middle of wing; veins R_4+5_ and M_1+2_ confluent for short distance and very strongly divergent more distally; hind wing without r-m crossvein, veins R_4+5_ and M_1+2_ confluent for short distance and divergent beyond; metathoracic leg without cucullate femoral setae, tibia without cucullate setae in row I. An additional similarity of the new genus and *Thuris* Funkhouser is presence of about eight cucullate setae on the first tarsomere of the metathoracic leg, and the dark markings on and around the apical veins of the forewing. Some treehoppers that have scoli on the mesonotum, metanotum, or both (e.g., *Alchisme* Kirkaldy) have shriveled, vestigial scoli in the adults underneath the pronotum. The absence of vestigial scoli cannot be inferred to mean that the nymph lacks thoracic scoli. Nevertheless, taken together with the absence of dorsally paired processes or fossae on the abdomen, it is more likely that the nymph, unknown, may be unarmored, as is the nymph of *Thuris* (McKamey and Porter 2016). Until now, the tribe was composed of one genus and two species (McKamey and Porter 2016). The new genus is easily distinguished from *Thuris*, which has a strongly keeled pronotum posteriorly. Bolivia represents a new country record for the tribe.

#### Flynnia fascipennis

Taxon classificationAnimaliaHemipteraMembracidae

(Funkhouser) comb. n.

Figs 5–9 , 10–12

Ophiderma fascipennis Funkhouser, 1919: 274 [sp. n.].

##### Description of male.

Length including wings in repose 3.9 mm, maximum width 1.8 mm. Pronotum, wings, and leg features as described for genus. Pronotum black with small white spot along the lateral margin and white V-shaped band before apex (Fig. 6). Male genitalia (Fig. 11) . Pygofer with lateral plate separated; subgenital plates fused basally to about mid length; styles hooked distally, recurved anterolaterally; aedaeagus U-shaped, shaft weakly expanded, flat anteriorly, margins and surface without dentae or serrations, gonopore posterior.

Female unknown.

##### Material examined.

*Ophiderma fascipennis* male holotype, BOLIVIA (USNM). *Thuris binodosus* (Goding) holotype (USNM). Unfortunately the locality label of *F. fascipennis* lacks precision, simply stating “Bolivien” (Fig. 9). One male, ECUADOR: Orellana Prov., Reserva Etnica Waorani, 1 km S Onkone Gare Camp Transect Ent. 2163m, 3-Feb-1995, 00°39'25.7"S, 076°27'10.8"W. T.L. Erwin et al. Fogging terre firme forest. Lot#954 (USNM). One male, same data as previous except 6-Jul-1995 and Lot#1115 (USNM).

### Subfamily Smiliinae, *incertae sedis*

#### Antianthe atromarginata

Taxon classificationAnimaliaHemipteraMembracidae

(Goding) comb. n.

Figs 13–16

Cyrtolobus (Atymna) atromarginata Goding, 1928: 137 [sp. n.].
Atymna atromarginata ; Plummer 1938: 237

##### Material examined.

Holotype (USNM). Abdomen, left forewing, and both extended humeral angles missing. The species was described from Ecuador, Guayas Prov., Cerro Manglaralto (Fig. 16).

Goding’s holotype is probably a junior of *A. expansa* (Germar), which is also recorded from Ecuador. Wallace (2011) removed *Antianthe* Fowler from Smiliini. The holotype labels (Fig. 16) erroneously spell the species as “*marginata*” but the species description and locality match the published name “*atromarginata*.” The same mistake (and same missing prefix) was made with the holotype (USNM) labels of *Cymbomorpha atromaculata* Goding.

**Figures 13–16.**
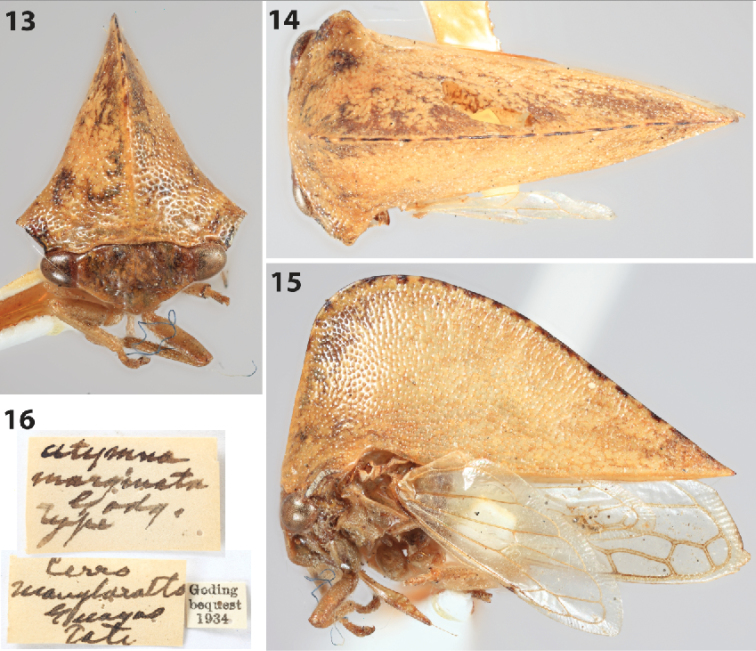
*Antianthe atromarginata* (Goding), comb. n., holotype. **13–15** Habitus in anterior, dorsal, and lateral views, respectively. Note that the extended humeral angles are broken off **16** Holotype labels.

## Supplementary Material

XML Treatment for Amastris pilosa

XML Treatment for Polyglyptini

XML Treatment for Flynnia

XML Treatment for Flynnia fascipennis

XML Treatment for Antianthe atromarginata
